# Behavioral Features in Phelan–McDermid Syndrome: Characteristics and Genetic and Metabolic Contributions in a Cohort of 56 Individuals

**DOI:** 10.3390/genes17020202

**Published:** 2026-02-08

**Authors:** Emily Payne, Bridgette A. Moffitt, Lindsay M. Oberman, Laura Beamer, Sujata Srikanth, Lauren Nicole Cascio, Kelly Jones, Lavanya Jain, Rini Pauly, Melanie May, Cindy Skinner, Carrie Buchanan, Barbara G. DuPont, Rebekah R. Martin, R. Curtis Rogers, Katy Phelan, Sara M. Sarasua, Walter E. Kaufmann, Luigi Boccuto

**Affiliations:** 1 Healthcare Genetics and Genomics Program, School of Nursing, Clemson University, Clemson, SC 29634, USA; 2 National Institute of Mental Health, National Institutes of Health, Bethesda, MD 20892, USA; 3 Greenwood Genetic Center, Greenwood, SC 29646, USAdupont@ggc.org (B.G.D.);; 4 Department of Biomedical Engineering, Cleveland Clinic Research, Cleveland, OH 44195, USA; 5 Genetics Department, Florida Cancer Specialists & Research Institute, Fort Myers, FL 33905, USA; 6 Department of Human Genetics, Emory University School of Medicine, Atlanta, GA 30322, USA; 7 Department of Neurology, Boston Children’s Hospital, Boston, MA 02115, USA

**Keywords:** Phelan–McDermid syndrome, *SHANK3*, autism spectrum disorder, metabolomics

## Abstract

**Background/Objectives**: Phelan–McDermid syndrome (PMS), caused by either chromosome 22q13.3 deletions or pathogenic/likely pathogenic variants in the *SHANK3* gene, is a rare neurodevelopmental disorder. Behavioral issues greatly impair the quality of life for affected individuals and their families. This genotype–phenotype study intended to further characterize key behavioral features and their genetic and metabolic correlates in PMS. **Methods**: We conducted a cross-sectional analysis of data on 56 individuals with PMS. Autistic and related behaviors were assessed with the Autism Diagnosis Interview—Revised (ADI-R) and adaptive behavior skills were assessed with the Vineland Adaptive Behavior Scales-Third Edition (Vineland-3), both covering multiple aspects of communication, socialization and abnormal behaviors. Genetic diagnostic information on deletions or pathogenic variants was supplemented with the sequencing data of nine candidate genes on 22q13.3. Metabolic data were obtained using the Biolog Phenotype Mammalian MicroArray plates (PM-M). **Results**. Every subject in the cohort presented either prominent autistic behavior or adaptive behavior impairment, 55.4% of them meeting the ASD cutoff in every ADI-R domain and 92.9% scoring in the lowest level of adaptive behavior (range of 20–70). Individuals with *SHANK3* variants had lower adaptive behavioral skills than those with 22q13 deletions regardless of deletion size, while genomic parameters were largely unrelated to ADI-R scores. Metabolic profiling identified unique profiles of individuals with PMS compared with controls, while distinct profiles distinguished those who met or did not meet the ADI-R ASD cutoff. Cluster analyses revealed groups of individuals with ASD and other clinical features. **Conclusion.** This study highlighted the importance of *SHANK3* in adaptive behavioral skills and uncovered potential metabolic biomarkers of therapeutic relevance.

## 1. Introduction

Phelan–McDermid syndrome (PMS) (OMIM*606232) is a rare neurodevelopmental disorder with a variable genotype and a phenotype that differs widely among individuals with PMS [1,2,3]. The clinical presentation of PMS may include developmental delay, moderate to severe intellectual disability, absent or severely delayed speech, neonatal hypotonia, autism spectrum disorder (ASD) and other problem behaviors, seizures, sleep disturbances, minor dysmorphic features, fleshy hands, and gastrointestinal issues, among others [1,2]. PMS has an estimated prevalence of 1 in 30,000, but it is most likely underdiagnosed due to the variable symptomatology [3,4]. Presently, there is no cure for PMS, so a multidisciplinary approach of supportive care to improve quality of life and reduce complications is the typical management [2,5]. The genetic cause of PMS can either be from deletions within chromosome 22q13.3 or pathogenic/likely pathogenic variants in *SHANK3* [2,6,7,8]. Most deletions are terminal and usually include *SHANK3*, but some individuals have been shown to exhibit PMS symptoms without involving the *SHANK3* gene [9]. This gene encodes SH3 and multiple ankyrin repeat domains 3 (SHANK3), a scaffolding protein located in the postsynaptic density of excitatory glutamatergic synapses, which is necessary for the formation and stability of postsynaptic terminals by connecting membrane-bound receptors to actin. The loss of or mutations in *SHANK3* disrupt the signal transmission of several excitatory synapses across the central nervous system. For this reason, *SHANK3* is one of the most commonly altered genes in cases of isolated ASD and is considered to be the primary candidate gene for the observed neurological and behavioral symptoms associated with PMS [1,2,6,7,8].

Regarding behavioral problems that are commonly reported in individuals with PMS, typical features of ASD are highlighted, such as restricted and repetitive behaviors, difficulties with social and communication skills, self-injury, impulsive behavior and, in line with developmental delay, impaired adaptive behavior [1,2,3,6,8,10,11,12,13]. The percentage of individuals with PMS exhibiting autism-like traits and other behavioral abnormalities has been reported to range from around 50% to over 90%, and 30% to over 80% of individuals with PMS have ASD diagnoses [3,6,8,10,11,13].

While behavioral issues have been reported in large percentages of individuals with PMS, gaps and inconsistencies remain in the current literature. Although various studies have included some aspect of behavior in the analysis [6,7,8,11,12,13,14], there are fewer studies with a sole focus on the behavioral characteristics of PMS [4,10,15,16]. Some argue that differences seen in behavioral features among individuals with PMS are based on characteristics such as age [11,12,13], deletion size [7,8,10], 22q13.3 deletion vs. *SHANK3* variant [3,4,6], and cognitive level [3,4,6,10,15], while others argue that these factors are unlikely to play a role in these behavioral differences [4,8,11,15]. Individuals with PMS have been characterized as having a unique behavioral phenotype with a complex genetic contribution to neurobehavioral impairments. Contributions from both deleted and preserved genes in the *SHANK3* region highlight the complexity of genetic contributions to the behavioral phenotype of PMS [16].

There is a lack of research into the metabolomics of individuals with PMS. The technique used in this paper to study metabolism has been applied in previous investigations examining underlying metabolic pathways in ASD and other genetic conditions, such as PMS [17,18,19,20,21,22]. With a high prevalence of ASD in individuals with PMS, this technique was a natural choice to study the PMS population.

The gaps in knowledge surrounding the clinical, genetic, and metabolic factors related to the behavioral features of PMS prevent the ability to diagnose accurately or treat abnormal behaviors in this population. A deeper understanding of the behavioral phenotype of PMS is important to aid in the clinical assessment, diagnosis, and treatment of PMS. For a better understanding of behavioral issues in PMS, there have been recommendations to increase the use of in-person direct assessments combined with validated, standardized methods of assessment, particularly those able to assess individuals with intellectual disability accurately [10,23]. Suggested treatments include intensive behavioral interventions with constant repetition to see progress with behavioral issues in individuals with PMS [5].

Two articles studying this same cohort of individuals with PMS looked specifically at the genetic, clinical, and metabolic contributions to sleep disturbances [22] and seizures [19]. A prior study with minimal overlap in participants conducted by the same investigators focused on autistic behavior in PMS, its characteristics and its relationship with genotypic features [16]. The present paper aims to expand this work and characterize behavioral features in PMS along with any genetic or metabolic contributions to them. This study also aims to look across the behavioral features, sleep disturbances, and seizures in this population of individuals with PMS to determine if any commonalities are found.

## 2. Materials and Methods

### 2.1. Participants in the Study

Participants were recruited through the Phelan–McDermid Syndrome Foundation (PMSF) and the Greenwood Genetic Center (GGC), Greenwood, South Carolina. The research was approved by the Self Regional Health System Institutional Review Board (Pro0057738). All parents or guardians of the participants were provided with written informed consent. By signing such consent, they acknowledged that the study was not bringing harm to the participants and that the deidentified study findings were going to be disseminated among the scientific community and the PMS families and Foundation. Participants with PMS were divided into four groups: 0–3 years, 4–10 years, 11–17 years, and 18 years and older, according to their age at the time of enrollment in the study. These age groups correspond to infants, children, adolescents, and adults.

### 2.2. Clinical and Genetic Information

Laboratory reports obtained by medical records and the Phelan–McDermid Syndrome DataHub (PMS DataHub, https://pmsf.org/datahub/, accessed on 1 June 2016) showing either a chromosome 22q13.3 deletion or a pathogenic *SHANK3* variant were used to confirm PMS diagnoses in all participants. For 33 participants with 22q13.3 deletions, chromosomal breakpoints were reported using the hg38 build or were converted to the hg38 build using the UCSC LiftOver tool (RRID:SCR_018160) [24]. Another 14 participants had confirmed 22q13.3 deletions, but breakpoint data were unavailable. Deletion breakpoint data and variant presence have been previously published for each participant in the study [19].

Data on abnormal behavior were collected using the Autism Diagnostic Interview—Revised (ADI-R), while data on adaptive behavior (skills) were collected with the Vineland Adaptive Behavior Scales—Third Edition (Vineland-3) [25,26]. The ADI-R is an investigator-based interview in a semi-structured format for caregivers of adults or children for whom autism spectrum disorder (ASD) or other developmental disorders might be suspected. It focuses on questions of communication, social development, restricted and repetitive behaviors, and general behavior, and they can typically take 1.5 to 2.5 h to complete the 93 questions. The ADI-R has been described as reliable and valid for making the diagnosis of ASD. The ADI-R includes four domains: Reciprocal Social Interaction, Communication, Restricted and Repetitive Behaviors, and Development (abnormal development evident at or before 36 months), each with its own scoring range and individual threshold levels for an indication of an ASD diagnosis [25]. A higher score in a domain of the ADI-R typically indicates increased behavioral impairment rather than a lower score. The Vineland-3 measures adaptive behavior for ages ranging from birth to 90 years old, and the comprehensive interview form used in this study comprises 381 items [26]. The Vineland-3 includes four domains: Communication, Daily Living Skills, Socialization, and Motor, which are each composed of subdomains. Vineland-3 is scored in a Likert-type format, reflecting the frequency of a particular adaptive behavior/skill. It is considered a reliable and valid measure of adaptive behavior suitable for clinical, research, and educational purposes [27]. The standard score in each category has a mean of 100. The scoring range of Vineland-3 for each category is on a scale of 20–140: low (20–70), moderately low (71–85), adequate (86–114), moderately high (115–129), and high (130–140). Higher scores on the Vineland-3 represent higher levels of adaptive functioning. Scoring ranges for each category are the following: at each end, 20–70 indicate a low level of adaptive functioning and 130–140 indicate a high level of adaptive functioning, while scores in the middle indicate moderately low (71–85), adequate (86–114), and moderately high (115–129) levels of adaptive functioning. In this study, we restricted the analyses to standard scores of the Communication, Daily Living Skills, and Socialization domains as well as the overall Composite score.

### 2.3. Genetic Sequencing

Genetic sequencing of nine genes of interest (*SULT4A1* (OMIM*608359), *PNPLA3* (OMIM*609567), *ARHGAP8* (OMIM*609405), *hsa-miR-1249*, *ALG12* (OMIM*607144), *PIM3* (OMIM*610580), *MAPK9IP2/IB2* (OMIM*607755), *SHANK3* (OMIM*606230), and *RABL2B* (OMIM*605413) located on chromosome 22q13 was conducted from blood samples from 40 participants. Further details on the genetic sequencing, genes and functions, primers used, and bioinformatics tools have been previously published [19,22].

### 2.4. Lymphoblastoid Cell Lines

To obtain metabolic profiles, lymphoblastoid cell lines (LCLs) were established from peripheral blood samples collected by venipuncture from 43 individuals, using lymphocyte immortalization via Epstein–Barr virus [28]. LCLs were harvested in Sigma RPMI-1640 with 15% fetal bovine serum (FBS) from Atlanta Biological (Flowery Branch, GA, USA), 2 mM L-glutamine, 100 U/mL Ppenicillin, and 100 μg/mL streptomycin from Sigma-Aldrich (St. Louis, MO, USA).

### 2.5. Metabolic Profiling (PM-M)

A specialized technology called Phenotype Mammalian MicroArray (PM-M) plates, developed by Biolog (Hayward, CA, USA), was used to identify metabolic signatures of behavioral features of LCLs from 40 individuals with PMS and 50 controls. Control blood samples had been previously collected from neurotypical children, adolescents, young adults, and adults. Signed informed consents were collected for the controls as well, according to the study protocol approved by the Self Regional Health System Institutional Review Board (Pro0057738). Participants meeting the ASD cutoff value for all four domains of the ADI-R (N = 21) were compared with those not meeting the ASD cutoff value for all four domains of the ADI-R (N = 19) along with control LCLs (N = 50). The PM-M plates measure the cellular production of NADH (nicotinamide adenine dinucleotide, reduced form) in the presence of different compounds to assess metabolic activity. Microplates are utilized with diverse molecules, acting as energy sources (plates PM-M1 to M4) or as metabolic effectors (plates PM-M5 to M8). The list of all compounds, including controls, is provided in Table S1. A single compound is within each well, and a colorimetric redox dye chemistry monitors the production of NADH per well. Essentially, metabolic dysregulation is indicated through NADH production. The energy sources include carbohydrates, carboxylic acids, ketone bodies, and nucleotides in plate PM-M1 as well as amino acids (both alone and as dipeptides) in plates PM-M2 to M4. The metabolic effectors include ions in PM-M5 along with cytokines, growth factors, and hormones in PM-M6 to M8. More in-depth information on the methodology used for the metabolic profiling using PM-M plates has been published previously [17]. After normalizing against triplicates of empty plates, data were analyzed by comparing to 50 control LCLs using the R package PhenoMetaboDiff version 1.0.0 [29] and the opm R package version 1.1.0 in R Studio 2024.04.02 [30]. The goal was to identify significant responses to energy sources and effectors, specifically compounds differentially metabolized by participants and controls, as well as any significant abnormalities in metabolic pathways.

### 2.6. Statistical Analysis

A single-factor ANOVA assuming normality was employed to measure associations between outcomes (ADI-R or Vineland-3) and the different deletion size and age range groups. Two-sided *t*-tests assuming normality and equal variance were utilized to compare means of two groups, such as for scores from ADI-R and Vineland-3 between the two sexes, or between those who were verbal or nonverbal, or for comparing means for a specific age group or deletion size compared to a referent. The Mid-P exact 2-tailed test from OpenEpi was used for associations of categorical data, specifically for the ADI-R ASD cutoff values [31]. The effects of deletion size and age (independent variables) on the ADI-R ASD cutoff and the Vineland-3 composite scores (dependent variables) of the subjects with known deletion sizes (n = 33) were assessed using linear regression and logistic regression analyses in SPSS version 29.0 [32]. Linear regression, both simple and multivariate, was used for the Vineland-3 composite scores, while binary logistic regression was used for the ADI-R ASD cutoff. The metabolic profiling data were analyzed using non-parametric Mann–Whitney two-sided tests, with a cut-off of *p*-value ≤ 0.05, which was followed by applying the Benjamini–Hochberg correction (R method: p.adjust) for multiple testing (FDR). SPSS was utilized to perform hierarchical clustering to evaluate if participants could be grouped by genetic and clinical characteristics [32]. First, Ward’s method of hierarchical clustering was performed to determine a representative number of clusters by examining the resulting dendrograms. Then, K-means hierarchical clustering was applied using the determined number of clusters from Ward’s hierarchical clustering to examine how the participants cluster based on various genetic and clinical characteristics. Clustering was performed twice: first based on the total cohort and secondly based on those with known 22q13.3 deletion sizes only. The clinical characteristics in both rounds of clustering included age range, sex, verbal/non-verbal status, the presence or absence of seizures [19], the presence or absence of sleep disturbances [22], meeting/not meeting ADI-R’s cutoff for ASD, and obtaining or not a Vineland-3 composite score in the low category.

## 3. Results

### 3.1. Participants

A total of 56 individuals with PMS were included in the study. There were 25 males (45%) and 31 females (55%), ranging from 3 to 45 years of age (mean 14.07). Forty-seven of these 56 individuals (84%) carried 22q13 deletions: 33 with known and 14 with unknown deletion sizes. Of the 33 with known deletion sizes, a *SHANK3*-unrelated deletion was found in one individual (3%). The remaining nine individuals without 22q13 deletions had a confirmed pathogenic variant within *SHANK3* (16%). Table 1 describes the PMS cohort’s characteristics and the scores on both the ADI-R and Vineland-3.

### 3.2. Abnormal Behavior and Adaptive Behavior Skills in PMS

Detailed results for both assessment tools, the ADI-R and Vineland-3, including the percentage of those that did and did not meet ADI-R cutoff values, average scores, and the *p*-values of both the *t*-test and ANOVA across sex, age, speech abilities, deletion sizes and *SHANK3* variant groups can be found in Table S2.

### 3.3. ADI-R

**Figure 1:**
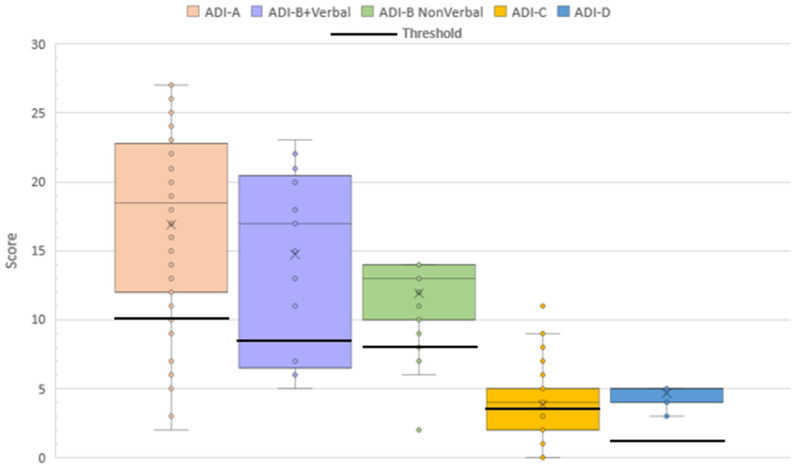
Distribution of ADI-R scores by category and ASD threshold.

The majority of the cohort met the threshold cutoff values for all components of the ADI-R: 55.4% for the ASD cutoff, 80.4% for the Reciprocal Social Interaction domain, 71.4% for the Verbal Social Communication domain, 94.3% for the Non-verbal Social Communication domain, 67.9% for the Restricted and Repetitive Behaviors domain, and 100% for the Development domain, showing that behavioral issues, according to the ADI-R, are a common occurrence in this cohort of individuals with PMS (Figure 1). Males scored significantly higher than females in each domain of the ADI-R, showing a higher impairment, except for Restricted and Repetitive Behaviors (S1). ADI-R domain scores did not generally vary by age with the exception that the youngest age group (0–3 years) scored significantly lower or less impaired when compared to the reference group (>18 years) on the Reciprocal Social Interaction domain (S1). The only significant difference between verbal and non-verbal individuals was on Restricted and Repetitive Behaviors with 85.7% of verbal individuals meeting the threshold and an average score of 4.81 compared to the 57.1% of non-verbal individuals meeting the threshold and an average score of 3.23 (*p* = 0.022) (S1).

### 3.4. Vineland-3

**Figure 2:**
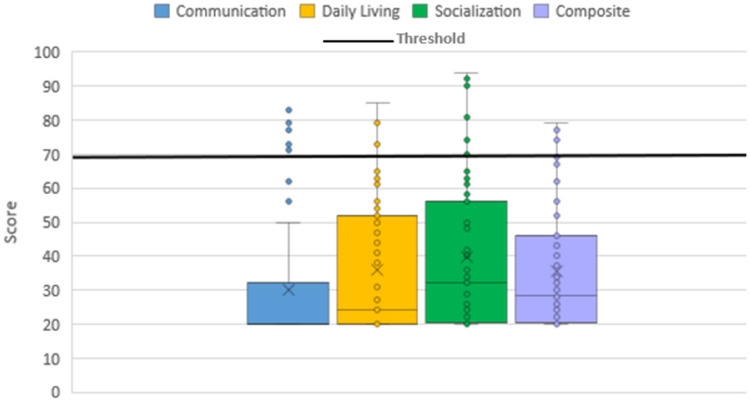
Distribution of Vineland-3 scores by category and the “low” adaptive behavior threshold.

The majority of the cohort fell below the score of 70 into the lowest adaptive level (“low”) in each domain and overall: 92.9% for the Composite score, 89.3% for Communication, 94.6% for Daily Living Skills, and 91.1% for Socialization (Figure 2). Thus, according to the Vineland-3, this PMS cohort is characterized by low levels of adaptive behavior. Males scored significantly lower in each category of the Vineland-3: Composite (*p* = 0.006), Communication (*p* = 0.017), Daily Living Skills (*p* = 0.024), and Socialization (*p* = 0.003). Using the >18 age group as the reference group for the *t*-test, age was statistically significant for all age groups in every category, according to ANOVA and *t*-test *p*-values, except for the 11–17-year-old-group in the Communication domain only (ANOVA): Composite (*p* = 6.32 × 10^−8^), Communication (*p* = 0.001), Daily Living Skills (*p* = 1.23 × 10^−9^), and Socialization (*p* = 1.72 × 10^−7^). Following a consistently negative trend in each category, the scores were highest in the 0–3-year-old group and lowest in the ≥18-year-old group. Non-verbal individuals scored significantly lower on both the Communication (*p* = 0.032) and Socialization (*p* = 0.044) domains (S1).

### 3.5. Regression Analysis

#### 3.5.1. Age, Deletion Size, and ADI-R ASD Cutoff

A binary logistic regression analysis was performed to investigate the potential effects of age and deletion size on the likelihood of meeting the ADI-R ASD cutoff. The model was non-significant (χ^2^ (2) = 2.66, *p* = 0.265). The model explains only between 7.7% (Cox and Snell R Square) and 10.5% (Nagelkerke R Square) of the variance in ADI-R ASD status but correctly classifies 75.8% of cases. The Hosmer and Lemeshow test suggested a moderately good fit to the data (χ^2^ (8) = 13.13, *p* = 0.107). Neither age nor deletion size were significant in the model as a predictor of meeting the ADI-R ASD cutoff: age (*B* = 0.09, *Wald* = 2.31, *p* = 0.128, *Exp* (*B*) = 1.10, 95% *CI* [0.97, 1.24]) and deletion size (*B* = 0.00, *Wald* = 0.00, *p* = 0.998, *Exp* (*B*) = 1.00, 95% *CI* [0.76, 1.31]). The equation of this model is ln(*p*/*p* − 1) = −0.692 + (0.093 × age) + (0 × deletion size) (Table 2).

#### 3.5.2. Age and Vineland-3 Composite Score

The overall simple linear regression model was statistically significant (F = (1,31) 24.245, *p* ≤ 0.001), suggesting that the model predicts Vineland-3 composite scores better than would be expected by chance. An R^2^ of 0.439 shows that 43.9% of the variance in Vineland-3 composite scores can be explained by age alone. The Durbin–Watson value fell within the desired range of 1.5–2.5 with a score of 2.003. The regression equation for this model is Vineland-3 composite = 63.576–1.937 (age), meaning that with one unit increase in age, we predict Vineland-3 composite scores to decrease by 1.937 units (Table 2).

#### 3.5.3. Deletion Size and Vineland-3 Composite Score

The overall simple linear regression model was statistically significant (*F* = (1,31) 7.649, *p* = 0.009), suggesting that the regression model predicts Vineland-3 composite scores better than would be expected by chance. The R^2^ = 0.198 shows that only 19.8% of the variance in Vineland-3 composite scores can be explained by deletion size alone. The Durbin–Watson value fell within the desired range of 1.5–2.5 with a score of d = 2.288. The regression equation for this model is a Vineland-3 composite = 51.440–3.145 (deletion size), meaning that with each one-unit increase in deletion size, we predict the Vineland-3 composite scores to decrease by 3.145 units (Table 2).

#### 3.5.4. Age, Deletion Size, and Vineland-3 Composite Score

The overall multiple regression model was statistically significant (*F* = (2,30) 17.812, *p* < 0.0010), suggesting that the model including the variables of age and deletion size predicts Vineland-3 composite scores better than would be expected by chance. The R^2^ = 0.543 shows that 54.3% of the variance in Vineland-3 composite scores can be explained by age and deletion size. The Durbin–Watson value fell within the desired range of 1.5–2.5 with a score of 2.112. The regression equation for this model is Vineland-3 composite = 70.187 − (1.751 × age) − (2.323 × deletion size). Both independent variables, age and deletion size, are statistically significant for the model prediction with *p* ≤ 0.001 and *p* = 0.014, respectively (Table 2).

### 3.6. 22q13 Deletion vs. SHANK3 Variant

In general, there was no significant difference in ADI-R scores between individuals with 22q13 deletions and those with *SHANK3* variants with the one exception that those with deletions had a significantly higher, or more severe, score than those with *SHANK3* variants on the ADI-R Development domain (*p* = 0.045) (S1). Among the deletion size subgroups, those with 3–6 Mb deletions were significantly more severe on the ADI-R Development than those with smaller or larger deletion sizes. Scores on the other ADI-R domains were generally similar across deletion size (Table 2 and Table S1).

For the Vineland-3, apart from the > 6 Mb deletion size group in the Socialization domain (*p* = 0.047), there was no significant difference among the deletion size groups in any of the Vineland-3 domains. Individuals with *SHANK3* variants scored significantly lower, or more severe, in each domain and overall than individuals with 22q13 deletions: Composite (*p* = 0.0001), Communication (*p* = 0.002), Daily Living Skills (*p* = 1.251 × 10^−7^), and Socialization (*p* = 0.040) (S1).

### 3.7. Behavior and Variants in Nine Candidate 22q13 Genes

**Figure 3:**
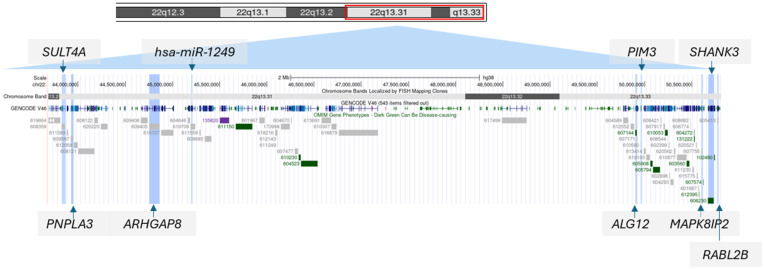
Nine candidate genes in the 22q13.3 region.

We sequenced nine genes located in the 22q13.3 region with the purpose of identifying additional variants that might contribute to the PMS phenotype in addition to the *SHANK3* pathogenic variants or deletions in 22q13 (Figure 3). The nine 22q13.3 genes were targeted due to their loci, expression patterns, roles of their proteins, and connections with some features reported in PMS according to the literature. Complete sequencing data were available on 40 individuals out of the 56. Potentially deleterious variants were identified in the following genes: *PNPLA3*, *ARHGAP8*, *ALG12*, *SHANK3*, and *RABL2B*. The list of variants is reported in Table S3. The classification of the variants was based upon an allele frequency less than 0.1 according to gnomAD, a CADD threshold >16, and literature information on function. Twenty-eight individuals (70%) had at least one deleterious variant reported [19]. Of those with a deleterious variant, 15 (54%) individuals met the ADI-R ASD cutoff and 26 (93%) scored in the low adaptive level for the Vineland-3 composite score (S2). Twenty-two individuals reported 22q13.3 deletions with 13 (59%) meeting the ADI-R ASD cutoff and 20 (91%) scoring in the low adaptive level for the Vineland-3 composite score. The one individual with a deleterious variant in *SHANK3* did not meet the ADI-R ASD cutoff and fell in the moderately low range of the Vineland-3 for adaptive behavior. There were no statistically significant associations between the presence of a deleterious variant and behavioral features according to the Vineland-3 or the ADI-R (S2). Additionally, there were no statistically significant associations between the presence of one deleterious variant versus the presence of more than one deleterious variant and behavioral characteristics measured by the Vineland-3 or the ADI-R (S2) [19].

### 3.8. Behavioral Features and Metabolic Profiles

Metabolic profiles were assessed using the Biolog PM-M platform in 40 PMS and 50 control LCLs. The list of wells with significant differences (*p* < 0.05) for those meeting the ADI-R ASD cutoff and those not meeting the ADI-R ASD cutoff is shown in Table S4. Of the combined 776 wells, the individuals meeting the ASD cutoff had 84 (10.8%) wells with levels of NADH production significantly different from controls compared to the individuals not meeting the ASD cutoff with 24 (3.1%) wells with levels of NADH production significantly different from controls. This includes 30 wells with increased NADH production and 54 with decreased NADH production in the group meeting the ASD cutoff compared to 5 with increased production and 19 with decreased production in the group not meeting the ASD cutoff (Table S4). Thus, those meeting the ASD cutoff showed higher numbers of differentially metabolized compounds than those not meeting the ASD cutoff of the ADI-R.

**Figure 4:**
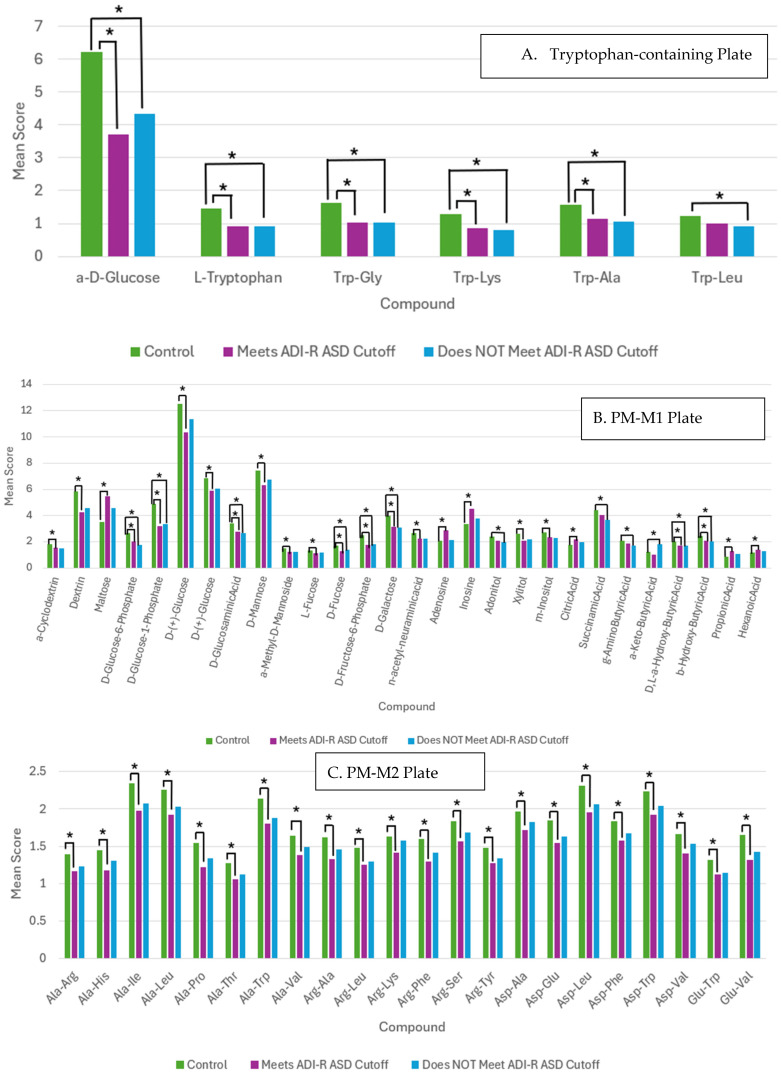

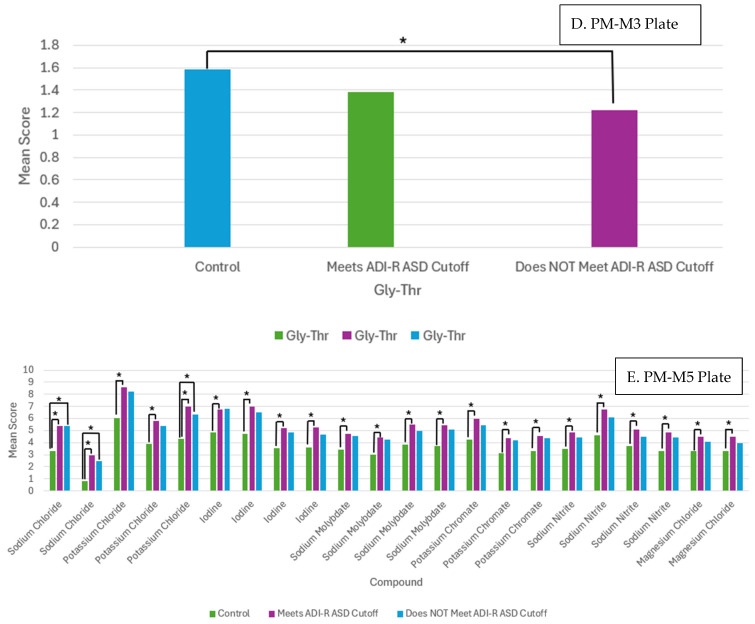
Mean scores of significant wells comparing subjects who meet ADI-R ASD cutoff, subjects who do not meet ADI-R ASD cutoff, and controls. (**A**) Tryptophan-containing plate. (**B**) PM-M1 Plate. (**C**) PM-M2 Plate. (**D**) PM-M3 Plate. (**E**) PM-M5 Plate. * indicates *p* < 0.05.

Similar metabolic profiles were seen between both groups for the tryptophan-containing plate with five of the same compounds significantly decreased compared to controls: a-D-glucose, L-tryptophan, Trp-Gly, Trp-Lys, and Trp-Ala (Figure 4A). In the PM-M1 plate, eight of the significant wells in the non-ASD cutoff group were also significantly different from the controls in the ASD cutoff group for decreased NADH production; these wells included various carbohydrate and carboxylic acid compounds such as D-glucose-6-phosphate, D-glucose-1-phosphate, D-glucosaminic acid, D-fucose, D-fructose-6-phosphate, D-galactose, D, L-a-hydroxy-butyric acid, and b-hydroxy-butyric acid (Figure 4B). However, the ASD cutoff group had 16 additional significant wells not found in the non-ASD cutoff group with all but six wells showing a decrease in NADH production compared to controls. The high number of significant wells in both PMS groups indicates a reduced utilization of main energy sources, including carbohydrates (simple and phosphorylated sugars) and carboxylic acids, which are important for energy production. The ASD cutoff group also had a significant reduction in the energy produced in some wells containing nucleotides not found in the non-ASD cutoff group, such as adenosine and inosine. While both groups show an overall decrease in the metabolism of these energy sources, the ASD cutoff group had a large number of additional wells with a decreased utilization, such as dextrin, D-mannose, and L-fucose, indicating a potential disruption in energy-producing metabolic pathways.

One significant difference between the ASD cutoff group and the non-ASD cutoff group was with the PM-M2 plates (Figure 4C). The ASD cutoff group had 25 significant wells compared to controls, while the non-ASD cutoff did not have any significant wells. All of these wells showed a decreased utilization of the various amino acid dipeptides in individuals fully meeting the ASD diagnosis cutoff with a relatively high representation of the amino acids alanine (10 wells) and arginine (6 wells) (Table S4). For one well from the PM-M3 plate, Gly-Thr, the non-ASD cutoff group showed a significant reduction in NADH production compared to the control, which was not shown by the ASD cutoff group (Figure 4D). The decreased utilization of amino acids as energy sources, more evident in the cohort meeting the ASD cutoff, suggests potential disruptions in the pathways regulating protein synthesis and may affect the cellular response to metabolic distress.

The PM-M5 plate, with various ions that function as metabolic effectors, had some similarities and differences between both PMS groups (Figure 4E). Five wells showed significantly different levels of NADH production compared to controls in both the ASD and non-ASD cutoff groups, mainly with an increase in NADH production, including specific concentrations of sodium chloride, potassium chloride, and sodium tungstate. However, compared to the individuals who did not meet the diagnosis cutoff, the individuals who did meet the cutoff for ASD diagnosis had an additional 23 wells, including various concentration levels of ionic species such as potassium chromate, sodium nitrate, and magnesium chloride, almost all with an increase in NADH production (Table S4). This increased response to exposure to ionic species in cells from individuals who met the ASD cutoff suggests a potentially altered homeostasis of intracellular ion concentrations with repercussions on susceptibility to changes in membrane potential.

### 3.9. Comparison of Behavioral Features, Sleep Disturbances, and Seizures in the PMS Cohort

In addition to delineating behavioral features of PMS and their molecular and metabolic correlates, this study integrated behavioral data with that previously published on sleep disturbances [22] and seizures [19]. Heat maps and hierarchical clustering analysis were performed to determine if individual groupings were discernable in the cohort on the basis of genetic or clinical characteristics. Heat maps of the genetic and clinical characteristics of the cohort can be found in Table S5. Descriptive characteristics such as age, sex, and deletion sizes are in green and white. At the same time, behavioral features, sleep disturbances, and seizures data are shown in red and blue for the presence and absence of the characteristic, respectively. Each heatmap is meant to be viewed as a standalone map where each row represents one individual in the cohort. Hierarchical clustering was performed twice for the cohort: the first included only individuals with 22q13.3 deletions of known size (n = 33) (Table S4), and the second included the full cohort of 56 individuals (Table S5). Three clusters were chosen for both rounds based on the resulting dendrograms from Ward’s clustering (Tables S6 and S7). We found that sleep disturbances contributed significantly to the generation of clusters when including only subjects with known deletion sizes but not when including the entire cohort. Seizures significantly contributed to the generation of clusters in both those with known deletion size and the entire cohort of 56 individuals. While differences in seizures, sleep disturbances, and behavioral features were seen across clusters, no discernable pattern was observed among these three clinical characteristics.

## 4. Discussion

This study described behavioral characteristics in a cohort of 56 individuals with PMS, specifically autistic or autism-related behaviors (using the ADI-R) and adaptive behavior skills (using the Vineland-3). It also investigated the association of the behavioral features with the participants’ individual genetic and metabolic profiles. The entire cohort met the ASD threshold of at least one domain of the ADI-R. Overall, 3 out of 56 individuals (5%) met only one domain, while 6 (11%) met two domains, 16 (29%) met three domains, and 31 (55%) met all four domains (Reciprocal Social Interaction, Social Communication, Restricted and Repetitive Behaviors, and Development). Over half of the individuals in this cohort met the diagnostic cutoff for ASD, which is less than the findings of Soorya et al. [8] and Schon et al. [3] in which a clinical diagnosis of ASD was found in 84% and 57–79% of individuals, respectively; however, this proportion is higher than the results of Sarasua et al. [7] and Sarasua et al. [13] with 26% and 31% of individuals with parent-reported ASD, respectively. In our prior investigation [16], an analysis of ADI-R domains applying the DSM-5 framework, we found that 90% of individuals with PMS met the ASD diagnostic criteria in social communication but only 55% on restricted, repetitive patterns of behavior, interests, or activities.

The entire cohort scored in the low or moderately low adaptive levels overall and on every domain of the Vineland-3 (Composite, Communication, Daily Living Skills, and Socialization), agreeing with previous findings [12,16]. As in other neurodevelopmental disorders [33], most participants scored in the low category of adaptive behavior, suggesting that Vineland-3 might not be ideal as an assessment tool for better characterizing adaptive skills in individuals with PMS. In fact, a recent analysis of longitudinal trajectories of adaptive skills in PMS, using the Vineland-II, showed that standard scores decreased/remained constant for all domains. However, subdomain growth scale values [33] demonstrated small gains in the Communication and Daily Living Skills but not in the Socialization domain [34].

There was a statistically significant difference between males and females for scores on both the ADI-R and Vineland-3, in contrast to the findings of Soorya et al. [8], in which there were no differences in autism severity or adaptive behavior according to sex. In this study, males consistently scored higher (i.e., more impairment) in every category of the ADI-R. Males also scored significantly lower in every category of the Vineland-3, showing that based on the two assessment tools, autistic and related behaviors and adaptive behavior impairment are more common and more severe in males with PMS. Abnormal behavior, as measured by the ADI-R, did not differ with age. However, in almost every category/age group combination of factors influencing the Vineland-3, age was significantly associated with adaptive behavior impairment, which agrees with Burdeus-Olavarrieta et al. [12] and the recent longitudinal study by Srivastava and colleagues [34]. These findings are in line with the recently reported small gains over time in some adaptive behavior domains, which are detectable by growth scale values but not by standard scores [34]. Verbal individuals had significantly higher scores (more severe) in the ADI-R’s Restricted and repetitive behaviors domain compared to their nonverbal counterparts. However, the nonverbal cohort scored significantly lower (worse) in the Communication and Socialization domains of the Vineland-3, indicating that greater verbal skills may be associated with some more severe autistic features in PMS.

Overall, adaptive behavior, as measured by Vineland-3 scores, was more impaired in individuals with *SHANK3* variants than in those with 22q13 deletions, and among those with deletions, it was more impaired in individuals with larger deletions compared to those with smaller deletions. Meeting the ASD cutoff on the ADI-R was similar for those with *SHANK3* variants and those with 22q13 deletions, and increased deletion size did not affect the severity of the behavioral impairment, according to ADI-R, which is in agreement with the findings from Droogmans et al. [11]. The only exception observed involved the ADI-R Development scores, where individuals with 22q13 deletions had more severe scores than those with *SHANK3* variants.

This suggests that 22q13 deletions have a greater negative effect on the development of skills, particularly language, than *SHANK3* variants alone, while the core behavioral features appear to be associated with *SHANK3* disruption. Our results align with those from Soorya et al. [8], who observed that larger deletion sizes correlated with more severe behavioral phenotypes, and Landlust et al. [10], who found that smaller deletion sizes correlated with better levels of adaptive behavior skills [7,11]. However, individuals with *SHANK3* variants scored significantly lower on all domains of the Vineland-3, showing that adaptive behavioral skills might be more severe when *SHANK3* is directly affected when compared to individuals with 22q13.3 deletions of any size. This contrasts with the findings of Serrada-Tejada et al. [4] indicating that those with *SHANK3* variants scored better in adaptive behavior, specifically socialization. Our finding that deletion size does not contribute significantly to PMS’s ASD-related features supports a selective role for *SHANK3* in neurological and behavioral abnormalities, as previously suggested by Sarasua et al. [7] and Soorya et al. [8].

The regression analysis models for predicting Vineland-3 composite scores were significant for the independent variables of age and deletion size individually and combined, showing that these variables did contribute to the Vineland-3 composite scores in this cohort. These models showed that 19.8%, 43.9%, and 54.3% of the variation in Vineland-3 composite scores can be attributed to the independent variables of deletion size, age, and the combination of age and deletion size, respectively. Both independent variables showed a negative relationship with the composite score being lower as either age or deletion size increased. This supported the above-mentioned finding that as age increased, adaptive behavior scores decreased. While deletion size was not statistically significant for Vineland-3 scores in this cohort, the regression model results highlight the potential relationship between deletion size and adaptive behavioral skills. On the other hand, age and deletion size were not statistically significant in the regression model to predict the likelihood of meeting the ADI-R ASD cutoff in individuals with PMS. This supported the aforementioned findings that neither age nor deletion size were significant for ADI-R scores in this cohort.

There was a notable difference in metabolic profiles between individuals who met the ADI-R ASD diagnosis cutoff versus those who did not. Both PMS groups had unique metabolic profiles compared to the controls, particularly with the decreased utilization of energy sources such as carbohydrates and carboxylic acids, as well as the compounds found in the tryptophan-containing plate. The common decrease in the utilization of tryptophan as an energy source confirms that alterations in the metabolism of this amino acid are a consistent metabolic finding in neurodevelopmental disorders with autistic traits [17]. However, the ASD cutoff group also showed a significant decrease in amino acid utilization, which was not found in the non-ASD cutoff group. While both groups had an increased utilization of ionic species, the ASD cutoff group showed a significant increase in NADH production in 20 additional wells compared to the non-ASD group, possibly suggesting a higher sensitivity to perturbations of ionic concentrations or pH that might lead to altered membrane potentials. These distinct metabolic profiles indicate that in general, individuals with PMS appear to have a unique metabolic profile compared to controls for various energy sources and metabolic effectors. Additionally, individuals who met the ADI-R ASD cutoff also exhibit a unique profile when compared to those who did not meet the cutoff with broader alterations in pathways involving amino acids like alanine, arginine, aspartate, and glutamate as well as an increased metabolic response to exposure to ionic species. Therefore, metabolic profiles could indicate differences in behavioral phenotypes in individuals with PMS. Specifically, differences in metabolic profiles can potentially be used to identify alterations in biological pathways and biomarkers in individuals with PMS as well as potential molecular targets for drug development [19,22].

### Limitations of the Study

This investigation had some limitations. The wide age range (3–45 years) in our cohort may have introduced additional variation to our assessment. Cultural differences might have affected parental reporting of behavioral issues, especially behaviors regarding social interaction or daily life where cultural norms might vary. The study also relied upon convenience sampling, as this is a rare genetic disorder. Although the ADI-R and Vineland-3 are well-validated behavioral assessment tools, there are limitations in their application to populations like the PMS cohort included here. Normative scores of measures assessing cognition and adaptive skills tend to underestimate abilities as individuals with intellectual disability grow older because of their slower-than-average increases over time. This leads to both floor effects and measurement error when using standard scores, particularly when abilities are four standard deviations below average (standard score of 40) [33]. Considering that the Vineland-3 composite and domain mean standard scores were below 40 and that the majority of participants were in the low level group, many individuals may have had underestimated abilities [34]. Moreover, the potential lack of linearity of their scores may have affected their relationship with age and deletion size. The exclusive use of the ADI-R, without any evaluation of communication skills and implementation of a DSM-5 framework, may have led to overestimating social communication and interaction impairments [16].

Undoubtedly, additional measures of abnormal behaviors (e.g., disruptive behavior, anxiety) could have provided a more comprehensive view of the PMS behavioral phenotype. As previously reported by Jain et al. [19], due to differences in builds of the UCSC genome browser, the deletion breakpoints were based on estimates for six individuals. The analysis of variants is preliminary and requires confirmation using high-resolution whole-genome sequencing to confirm the findings of this study. While future studies could benefit from a larger sample size, a cohort of 56 individuals for a rare disorder is reasonably large. Remote data collection or international connections could increase participation in future studies. Future work could also benefit from comprehensive genomic analysis, including the addition of transcriptomics, epigenomics, and advanced multivariate methods, such as cluster analysis and principal component analysis, to identify behavioral phenotype subgroups and more refined genotype–phenotype correlations.

The utilization of LCLs as an in vitro model for metabolic profiles bears the intrinsic limitation of the possible interferences of the Epstein–Barr virus transfection with the cellular metabolism and the clonal nature (decreased variability and therefore representativeness) of LCLs. We planned to minimize this limitation by employing control LCLs so that the metabolic abnormalities eventually detected between PMS and control cells could be linked with more confidence to the genetic abnormalities carried by the PMS specimens.

## 5. Conclusions

**Figure 5:**
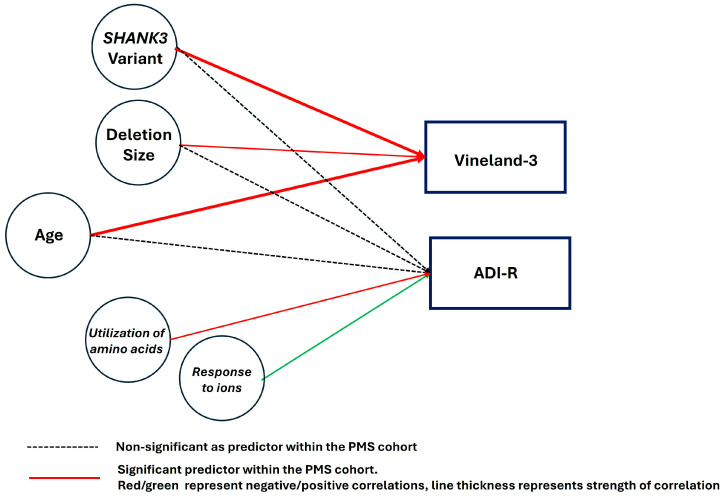
Summary of the main findings of the study.

This study showed that certain behavioral features, specifically lower levels of adaptive behavioral skills and ASD behaviors, are common in individuals with PMS (Figure 5). This supports the literature on the topic, confirming also previous reports of greater severity in males and lower standard scores on adaptive behavior skills in older subjects with PMS. Individuals with *SHANK3* variants scored significantly lower for adaptive behaviors compared to individuals with 22q13 deletions regardless of deletion size, suggesting that disruptions in *SHANK3* are the primary genetic contributor to the adaptive behavior impairments affecting their daily functioning. Nonetheless, regression models showing predictive effects of age and deletion size on Vineland-3 composite scores were significant, indicating that deletion size also plays a role in adaptive skills.

This study found unique metabolic profiles present in the PMS cohort compared to normal controls, particularly a reduced utilization of carbohydrates and carboxylic acids and increased utilization of ionic species for energy production. Additionally, a unique profile was identified when comparing individuals who met the ADI-R ASD cutoff to those who did not meet this cutoff—namely, a decreased utilization of amino acids for energy production and a more prevalent increase in energy production in the presence of ionic compounds compared to the non-ASD group. These unique metabolic profiles may suggest potential targets that may lead to the development of biomarkers for further investigation and novel drug development. Our cluster analyses failed to reveal unique subtypes including behavior; however, significant differences were observed for sleep disturbances and seizures. Altogether, the present study increased our understanding of the intersection between molecular, metabolic, behavioral, and other clinical features of PMS, paving the way for additional research in larger subject samples.

## Figures and Tables

**Table 1:** Descriptive characteristics of PMS cohort with ADI-R and Vineland-3 (cutoff values in parentheses).

	Characteristic		N	%	Mean	St. Dev.	Range
Total			56	100	n/a	n/a	n/a
Sex	Male		25	44.6	n/a	n/a	n/a
	Female		31	55.4	n/a	n/a	n/a
Age (years)	Total		56	100	14.1	8.5	3–45
	0–3		6	10.7	3	0	0–3
	4–10		12	21.4	6.8	2	4–10
	11–17		24	42.9	14.1	2	11–17
	>18		14	25	24.9	8.3	18–45
Speech	Verbal		21	37.5	n/a	n/a	n/a
	Nonverbal		35	62.5	n/a	n/a	n/a
Genetic Data	Deletion	Total	47	83.9	3.85 Mb *	2.7 *	0.06–8.45 *
		<3 Mb	16 *	48.5	1.3 Mb	1	0.06–2.98 Mb
		3–6 Mb	8 *	24.2	4.6 Mb	0.9	3.42–5.86 Mb
		>6 Mb	9 *	27.3	7.5 Mb	0.7	6.36–8.45 Mb
		Unknown Size	14	29.8	n/a	n/a	n/a
		*SHANK3*-unrelated	1 *	3.0	n/a	n/a	n/a
	*SHANK3* Variant		9	16.1	n/a	n/a	n/a
ADI-R	ASD Cutoff	Total	56	100	n/a	n/a	n/a
		Meets	31	55.4	n/a	n/a	n/a
		Does Not Meet	25	44.6	n/a	n/a	n/a
	ADI-R Reciprocal Social Interaction	Total	56	100	16.9	7	2–27
		Meets Cutoff (10)	45	80.4	19.6	4.8	10–27
		Does Not Meet Cutoff	11	19.6	5.7	2.1	2–9
	ADI-R Verbal Social Communication Score	Total	21	100	14.7	6.2	5–23
		Meets Cutoff (8)	15	71.4	18.2	3.5	11–23
		Does Not Meet Cutoff	6	28.6	6	0.6	5–7
	ADI-R Non-Verbal Social Communication	Total	35	100	11.9	2.8	2–14
		Meets Cutoff (7)	33	94.3	12.3	2.1	7–14
		Does Not Meet Cutoff	2	5.7	4	2.8	2–6
	ADI-Restricted and Repetitive Behaviors	Total	56	100	3.8	2.3	0–11
		Meets Cutoff (3)	38	67.9	5	1.9	3–11
		Does Not Meet Cutoff	18	32.1	1.4	0.8	0–2
	ADI-R Development	Total	56	100	4.7	0.5	3–5
		Meets Cutoff (1)	56	100	4.7	0.5	3–5
		Does Not Meet Cutoff	0	0	n/a	n/a	n/a
Vineland-3	Composite	Total	56	100	35.6	17.9	20–79
		Low (20–70)	52	92.9	32.4	14.4	20–70
		Moderately Low (71–85)	4	7.1	77	2.2	74–79
	Communication	Total	56	100	30.2	18.4	20–83
		Low (20–70)	50	89.3	24.8	10	20–62
		Moderately Low (71–85)	6	10.7	75.7	4.8	71–83
	Daily Living Skills	Total	56	100	36.1	19.1	20–85
		Low (20–70)	53	94.6	33.7	16.8	20–66
		Moderately Low (71–85)	3	5.4	79	6	73–85
	Socialization	Total	56	100	39.7	20.6	20–94
		Low (20–70)	51	91.1	35.1	15.3	20–70
		Moderately Low (71–85)	5	8.9	86.2	8.4	74–94

* Based on the 33 with known deletion sizes.

**Table 2:** Regression analysis results for age and deletion size effects on the ADI-R ASD cutoff and Vineland-3 composite score.

Regression	Dependent Variable	Independent Variables	Model-Fit *p*-value	Correct Classification %	Cox and Snell R Square	Nagelkerke R Square	(Exp) B	Variable *p*-value
Binary Logistic	ADI-R ASD Cutoff	Age	0.265	75.8%	0.077	0.105	1.098	0.128
		Deletion Size					1	0.998
**Regression**	**Dependent Variable**	**Independent Variables**	**ANOVA *p*-value**		**F**	**R^2^**	**B**	
Simple Linear	Vineland-3 Composite	Age	**<0.001**		24.245	0.439	−1.937	
		Deletion Size	**0.009**		7.649	0.198	−3.145	
Multivariate Linear	Vineland-3 Composite	Age	**<0.001**		17.812	0.543	−1.751	
		Deletion Size					−2.323	

## Data Availability

Restrictions apply to the datasets. Requests for access to the datasets should be directed to Luigi Boccuto, lboccut@clemson.edu.

## Supplementary Materials

The following supporting information can be downloaded at https://www.mdpi.com/article/10.3390/genes17020202/s1, Table S1: List of Biolog compounds; Table S2: Data table; Table S3: Variants; Table S4: Cells of Biolog plates with significant differences; Table S5: Heat maps; Table S6: Cluster analysis of participants with known deletion size; Table S7: Cluster analysis of all participants.

## Abbreviations

The following abbreviations are used in this manuscript:

ADI-R	Autism Diagnostic Interview-Revised
ASD	Autism Spectrum Disorder
PMS	Phelan–McDermid syndrome
